# Strain diversity in *Mycobacterium avium* subsp. *paratuberculosis*-positive bovine fecal samples collected in Switzerland

**DOI:** 10.3389/fvets.2023.1154516

**Published:** 2023-04-26

**Authors:** Melina Rasper-Hössinger, Michael Biggel, Roger Stephan, Frauke Seehusen, Simone Scherrer

**Affiliations:** ^1^Institute of Veterinary Pathology, University of Zurich, Zurich, Switzerland; ^2^Institute for Food Safety and Hygiene, University of Zurich, Zurich, Switzerland; ^3^Section of Veterinary Bacteriology, Institute for Food Safety and Hygiene, University of Zurich, Zurich, Switzerland

**Keywords:** *Mycobacterium avium* subsp. *paratuberculosis*, MIRU-VNTR, cattle-type, sheep-type, fecal samples

## Abstract

*Paratuberculosis* or Johne’s disease is a chronic intestinal disease in domestic and wild ruminants. It affects global dairy economy and is caused by *Mycobacterium avium* subsp. *paratuberculosis* (MAP). The objective of this study was to analyze strain diversity in MAP-positive fecal samples by using a particular single nucleotide polymorphism (SNP) distinguishing between cattle (C-) and sheep (S-) type MAP and analysis of SNPs within *gyrA* and *gyrB* genes differentiating between Types I, II, and III. Moreover, mycobacterial interspersed repetitive unit and variable-number tandem repeat (MIRU-VNTR) analysis using eight established loci was performed. A total of 90 fecal samples from diseased animals presenting diarrhea and/or weight loss, originating from 59 bovine herds across 16 cantons of Switzerland were screened by PCR for the MAP-specific F57 and IS*900* genes and were further subtyped. 96.7% and 3.3% of the samples contained C- and S-type MAP, respectively. Ten INRA Nouzilly MIRU-VNTR (INMV) profiles, with a discriminatory index of 0.802, calculated based on 65 epidemiological independent genotypes, were detected: INMV 1 (33.8%), INMV 2 (23.1%), INMV 6 (16.9%), INMV 9 (9.2%), INMV 116 (4.6%), INMV 3 (3.1%), INMV 5 (3.1%) and INMV 72 (1.5%), including two novel INMV profiles, namely INMV 253 (3.1%; S-type III) and INMV 252 (1.5%; C-type). INMV 1, INMV 2, and INMV 6 comprised almost 75% of the F57- and IS*900-*positive samples. Typing data from 11 herds suggest that there are some herds with intra-herd diversity of genotypes. The results of this study indicate a heterogeneity of MAP in Switzerland.

## 1. Introduction

*Mycobacterium avium* subsp. *paratuberculosis* (MAP) is an important pathogen that causes chronic, progressive granulomatous enteritis called *paratuberculosis* (Johne’s disease). The disease was first reported in cattle and has spread worldwide, especially in the bovine population. The infection in small ruminants is also described globally in many goats and sheep (1). MAP is thought to be introduced in ruminant herds through trading of subclinically infected animals, although wildlife reservoirs are also thought to play a role in spreading the bacteria to livestock (2). To date no cure for diseased animals with chronic weight loss and decreased productivity exsists. Therefore, *paratuberculosis* has a strong impact on the global dairy economy. So far, MAP was neither proved nor excluded with confidence to play a causal role in the etiology of Crohn’s disease (3), however, a possible link to Crohn’s disease in humans has been suggested (4).

Two major lineages, which were named according to the host species from which they were first isolated, are called “Sheep-type” (S-type/Type I and Type III) or “Cattle-type” (C-type/Type II) strains (5, 6). C-type strains include also bison-type (B-type) isolates, which are based on differences in the IS*1311* insertion sequence (7). It has been recorded that MAP isolates belonging to different lineages are not host-specific (8, 9). Various molecular techniques have been used for investigating genetic diversity among MAP, such as restriction fragment length polymorphism (10), short-sequence repeat and pulsed-field gel electrophoresis typing (11), mycobacterial interspersed repetitive unit and variable-number tandem repeat (MIRU-VNTR) (12) or single nucleotide polymorphism (SNP)-based assay (13). For an ultimate resolution of the phylogenetic relationships between strains, whole-genome sequencing (WGS) of isolates can be used (9, 14–16), however requiring cultured isolates, which are laborious and time-consuming to obtain. Bovine *paratuberculosis* is caused mostly by C-type MAP and only in a few cases by S-type strains of MAP (1). In contrast to C-type strains, growth of S-type MAP strains in culture media is much slower and more fastidious (17, 18). C- and S-type MAP seem to differ in their virulence when looking at different host species potentially through adaption of their surviving and persisting capacities in response to the specific microenvironment of a host (6). Interspecies transmission of S-type strains was shown to occur in farms with close contact between co-grazing animals (19). In New Zealand beef cattle, S-type I MAP was detected more frequently than C-type MAP (19). The knowledge about the diversity of MAP strains may improve strain tracing.

In this study, the strain diversity of MAP in Switzerland was analyzed using a particular SNP distinguishing between C- and S-type MAP. Different SNPs within *gyrA* and *gyrB* genes were sequenced for differentiation between MAP Types I, II, and III. Moreover, MIRU-VNTR typing using eight established loci was performed in order to further characterize MAP.

## 2. Materials and methods

### 2.1. Fecal samples

A total of 90 fecal samples from 59 different herds originating across 16 cantons of Switzerland were collected between 2015 and 2021. The fecal samples were obtained from diseased cattle with diarrhea, weight loss, or in more than half of the cases from animals of different farms with other MAP-infected cattle. Only samples from actively MAP-shedding cattle in the subclinical or clinical stage were processed. Cattle examined represent 11 different breeds including 30% Limousin, 21.1% Holstein Friesian, 13.3% Simmental, 12.2% Angus, and 7.8% of each Charolais and crossbreeds, respectively. The rest of the cattle breeds (Blonde d ‘Aquitaine, Aubrac, Piemontese, Montbeliard, and Brown Swiss) included less than 10%. The average age of the examined cattle was 5 years with an interval between two and 11 years (Supplementary material). Eleven farms provided multiple samples of more than one animal per herd.

### 2.2. DNA extraction of fecal material

Deoxyribonucleic acid (DNA) extraction was performed by taking 5 g of fecal material using ID Gene Easy Preparation of Feces Samples (IDvet Genetics, Grabels, France) according to the manufacturer’s manual. For the mechanical lyzing step, the samples were homogenized twice by using TissueLyser II (Qiagen, Hilden, Germany) for 6 min at 30 Hz. DNA concentration and purity were determined using a NanoDrop 2000c Spectrophotometer (Thermo Fisher Scientific, Reinach, Switzerland) and stored at −20°C until use.

### 2.3. Identification and characterization of MAP

Each sample was investigated for the presence of the F57 gene and IS*900* using an in-house quantitative PCR (qPCR) (20). Samples were considered MAP-positive, if both two target genes F57 and IS*900* were detected.

Differentiation between C-type and S-type strains was determined based on PCR and restriction enzyme digestion of amplified PCR products, as described previously (13). Briefly, in a first step, an enzymatic restriction assay using BsmBI of a PCR product involving SNP3842359 (9, 13) was performed. A resulting PCR product of 528 bp indicated the presence of C-type MAP, whereas two PCR fragments of 312 bp and 216 bp demonstrated the presence of S-type MAP. Differentiation between Types I, II, and III was performed based on analysis of SNPs within *gyrA* and *gyrB* by Sanger sequencing (5).

Genomic DNA was analyzed using eight established MIRU-VNTR targets (12). Each reaction mixture contained HotStart *Taq* Master Mix Kit (Qiagen), Q-Solution (Qiagen; only for loci VNTR 10 and VNTR 32), 0.5 μM of each primer and 20 ng of purified genomic DNA in a final volume of 10 μl. PCR was performed for one cycle at 15 min at 95°C followed by 45 cycles at 95°C for 30 s, 60°C for 30 s, 72°C for 30 s, and a final step at 72°C for 10 min. The PCR amplification product was analyzed by capillary electrophoresis (QIAxcel, Qiagen) using a high-resolution cartridge (Qiagen), QX 15 bp-3 kb alignment marker (Qiagen) and QX 100 bp-2.5 kb size marker (Qiagen). Assignment of the length of PCR products was performed using QIAxcel ScreenGel Software version 1.3.0 (Qiagen). As a positive control, reference strain MAP ATCC 19698 was tested in each PCR run. INRA Nouzilly MIRU-VNTR (INMV) profiles were determined according to a previously described allele-calling table.^1^ The two new genotypic profiles detected were registered in the MAC-INMV database. Samples harboring an identical INMV type derived from the same farm were considered as related samples and were therefore not included in the panel of epidemiological independent samples. The distribution of the identified INMV profiles was calculated based on a subset of 65 epidemiologically unrelated samples. Simpson’s index of diversity was determined as described previously (21) referring to the selected sample panel of 65 epidemiological independent samples.

### 2.4. Analysis of phylogeny

**Figure 1:**
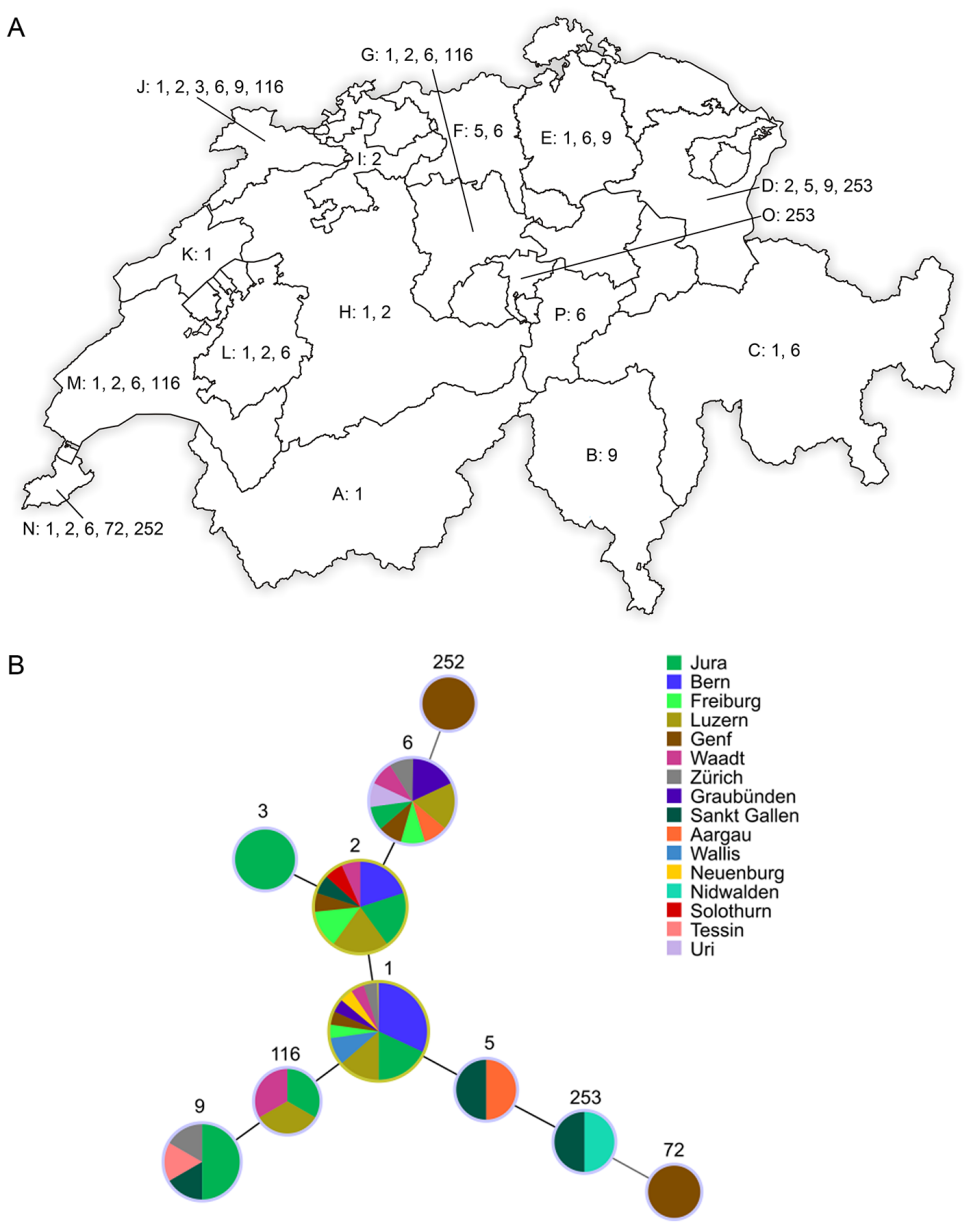
**(A)** Distribution of INMV types from F57- and IS*900*-positive fecal samples collected in 16 cantons of Switzerland: Valais (Wallis; A), Ticino (Tessin; B), Grisons (Graubünden; C), Saint Gallen (Sankt Gallen; D), Zurich (Zürich; E), Aargau (F), Lucerne (Luzern; G), Berne (Bern; H), Solothurn (I), Jura (J), Neuchâtel (Neuenburg; K), Fribourg (Freiburg; L), Vaud (Waadt; M), Geneva (Genf; N), Nidwalden (O), and Uri (P). Represented numbers correspond to the INMV types (INMV 1, 2, 3, 5, 6, 9, 72, 116, 252, and 253) observed in the respective cantons. INMV; INRA Nouzilly MIRU-VNTR. **(B)** Minimum spanning tree based on the comparison of INMV profiles identified in this study. Sizes of nodes reflect the number of epidemiological independent samples with a specific INMV profile and colors of nodes reflect the sample’s different geographical origin (canton) in Switzerland.

**Figure 2:**
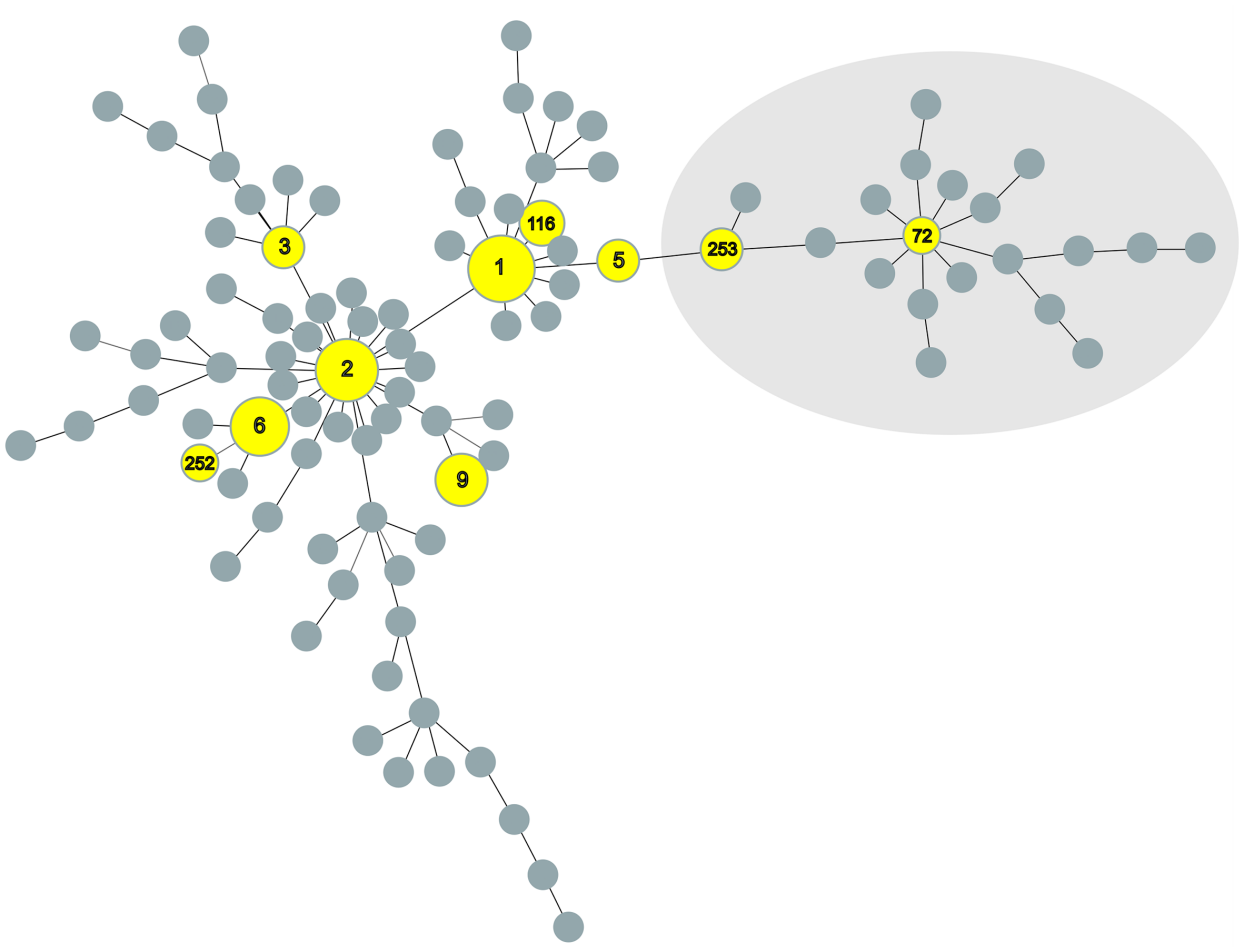
Minimum spanning tree of all *Mycobacterium avium* subspecies *paratuberculosis* INMV profiles registered at the MAC-INMV database (http://mac-inmv.tours.inra.fr/). Profiles that were also identified in this study or only in this study are colored in yellow. Grey nodes represent all INMV profiles registered at the MAC-INMV database. The size of these nodes reflects the number of epidemiologically unrelated samples harboring a specific INMV profile. INMV types 72 and 253, indicated in the light grey area, represent INMV profiles belonging to S-type *M. avium* subspecies *paratuberculosis* Type III. INMV; INRA Nouzilly MIRU-VNTR.

A minimum spanning tree (MST) was generated (Figure 1) based on data of the numerical code of eight VNTR loci of this study combined with all previously published data of MAP available (MAC-INMV database accessed on October 1st, 2022) using Phyloviz 2.0 (22) with the goeBURST algorithm (23). Another MST was created (Figure 2) including all INMV codes identified in Switzerland considering the geographical origin (canton) of the F57- and IS*900*-positive samples to visualize their geographical distribution.

## 3. Results

MAP F57 and IS*900* were successfully amplified for all 90 MAP samples with Ct-values ranging between 9.7 ≤ Ct ≤ 31.2 for IS*900* and 12.4 ≤ Ct ≤ 30.7 for F57, respectively, having an average Ct-value of around 21 for both targets (Supplementary material). Differentiation between C- and S-type MAP using a SNP assay revealed that 96.7% (*n* = 87) of samples belonged to C-type MAP and 3.3% (*n* = 3) belonged to S-type MAP. The analysis of SNPs within *gyrA* and *gyrB* genes allowed for the classification of C-type MAP samples into Type II and all three S-type MAP samples into Type III. Using MIRU-VNTR analysis, the 90 F57- and IS*900*-positive samples from 59 herds resulted in 10 INMV types (Table 1). In total, eight different known INMV types (INMV 1, INMV 2, INMV 3, INMV 5, INMV 6, INMV 9, INMV 72, and INMV 116) and two new profiles (INMV 252 and INMV 253) were identified (Table 1). The Simpson’s index of diversity, including the 65 epidemiological independent samples, resulted in 0.802.

**Table 1:** Total number of F57- and IS*900*-genes positive Swiss fecal samples classified into INMV types versus the number of epidemiological independent samples.

INMV type*	Number of tandem repeats at MIRU-VNTR loci**								Total number of samples	Number of epidemiological independent samples
	292	X3	25	47	3	7	10	32		
INMV 1	4	2	3	3	2	2	2	8	35	22
INMV 2	3	2	3	3	2	2	2	8	17	15
INMV 3	3	2	3	3	2	2	1	8	2	2
INMV 5	4	2	3	3	2	2	1	8	2	2
INMV 6	3	2	3	3	2	1	2	8	20	11
INMV 9	2	1	3	3	2	2	2	8	6	6
INMV 72	4	1	3	3	1	1	1	8	1	1
INMV 116	4	1	3	3	2	2	2	8	4	3
INMV 252	3	5	1	3	2	1	2	8	1	1
INMV 253	4	2	3	3	1	2	1	8	2	2

* MIRU-VNTR tandem repeats classified into INMV types (12).

** Eight established MIRU-VNTR loci classified numerically according to the MAC-INMV database (http://mac-inmv.tours.inra.fr/).

INMV; INRA Nouzilly MIRU-VNTR.

Considering the INMV profiles of the 65 epidemiological independent samples, INMV 1 (33.8%, *n* = 22), INMV 2 (23.1%, *n* = 15), and INMV 6 (16.9%, *n* = 11) were detected most frequently in the investigated F57- and IS*900*-positive samples. INMV 9 (9.2%, *n* = 6), INMV 116 (4.6%, *n* = 3), INMV 3 (3.1%, *n* = 2), INMV 5 (3.1%, *n* = 2), INMV 253 (3.1%, *n* = 2), INMV 72 (1.5%, *n* = 1), and INMV 252 (1.5%, *n* = 1) were found less frequently (Table 1).

The dominant INMV profiles 1, 2, and 6 were identified in at least eight cantons each (Figures 1A,B). INMV 9 was found in four cantons, INMV 116 in three cantons, INMV 5 and INMV 253 each in two cantons, INMV 3, INMV 72, and INMV 252 each in one canton (Figure 1B). In view of the herd distribution of the different INMV profiles, INMV 1 was found in 19, INMV 2 in 13, and INMV 6 in seven, INMV 9 in six, INMV 116 in three, INMV 5 and INMV 253 each in two, INMV 3, INMV 72, and INMV 252 each in one herd. In four of 11 herds, of which several samples per farm were analyzed, including two (*n* = 5), three (*n* = 2), or four to nine isolates (*n* = 4), two (*n* = 3) or four (*n* = 1) VNTR types were identified indicating intra-herd variability. Farms with observed intra-herd variability were located in region J (one herd with INMV 2 and INMV 6), in each region L and M (two herds with INMV 1 and INMV 6), and in region N (one herd with INMV 1, INMV 2, INMV 6 and INMV 252; Table 2).

**Table 2:** Geographic distribution of 90 F57- and IS*900*-positive fecal samples from 59 herds originating from 16 Swiss cantons (A–P) in relation to the number of herds, INMV profile, herds comprising a specific INMV profile, and number of samples.

Canton*	Number of herds	INMV profile**	Herds with INMV profile	Number of samples
A	2	1	2	3
B	1	9	1	1
C	3	1	1	1
		6	2	3
D	4	2	1	1
		5	1	1
		9	1	1
		253	1	1
E	3	1	1	1
		6	1	3
		9	1	1
F	2	6	1	2
		5	1	1
G	9	1	3	3
		2	3	4
		6	2	2
		116	1	1
H	10	1	7	11
		2	3	3
I	1	2	1	1
J	13	1	4	4
		2	2	2
		3	2	2
		9	3	3
		116	1	1
		2, 6	1	3 (2× INMV2, 1× INMV6)
K	1	1	1	1
L	3	2	2	2
		1, 6	1	9 (6× INMV 1, 3× INMV 6)
M	3	2	1	1
		116	1	2
		1, 6	1	4 (2× INMV 1, 2× INMV 6)
N	2	72	1	1
		1, 2, 6, 252	1	8 (3× INMV 1, 1× INMV2, 3× INMV 6, 1× INMV 252)
O	1	253	1	1
P	1	6	1	1

* Abbreviations of the 16 cantons: Valais (A), Ticino (B), Grisons (C), Saint Gallen (D), Zurich (E), Aargau (F), Lucerne (G), Berne (H), Solothurn (I), Jura (J), Neuchâtel (K), Fribourg (L), Vaud (M), Geneva (N), Nidwalden (O), and Uri (P).

** MIRU-VNTR tandem repeats classified into INMV types (12).

INMV; INRA Nouzilly MIRU-VNTR.

The creation of a MST-tree comparing the similarity of the INMV profiles, based on the repeat numbers of mini-satellites at eight defined MIRU-VNTR loci, showed a dispersed distribution of INMV types across the overall MAP population structure (Figure 2).

## 4. Discussion

The generated data provide an insight about the distribution of VNTR profiles in MAP positive fecal samples collected from diseased Swiss cattle of 16 different regions. MIRU-VNTR genotyping revealed the presence of at least 10 different MAP genotypes including both C- and S-type MAP. Moreover, up to four different genotypes were discovered in some of the investigated herds indicating a heterogeneity of MAP in Switzerland. A Simpson’s index of diversity of 0.802 underlines a diverse distribution of different MAP types, although an index of around 0.9 is considered as a minimum for a meaningful epidemiological survey (8). MIRU-VNTR profile INMV 1 was detected predominantly with over 33% of cases found in 10 different cantons, followed by INMV 2 and INMV 6 with 23.1 and 16.9%, respectively. Especially INMV 1 and INMV 2, to a lower extend also INMV 3, INMV 5, INMV 6, and INMV 9, are commonly found in Europe (12, 24), while S-type MAP (INMV 72) was only detected before in New Zealand and Spain (25). INMV 116 was reported to exist in Ireland and Germany (26, 27). The two new genotypes INMV 252 (C-type MAP; detected once) and INMV 253 (S-type MAP; detected in two cantons), were not observed in other countries so far. While extensive reports about the prevalence of S-type MAP strains in different countries are missing, probably due to its difficult cultivation, there are studies about differences of genomic organization between C- and S-type MAP showing that S-type MAP strains include more SNPs and show large rearrangements compared to C-type MAP strains (14, 28, 29).

Our results revealed that five of the eight loci studied (MIRU 292, MIRU X3, VNTR 25, VNTR 7, and VNTR 10) were found to be polymorphic, while two loci (VNTR 32 and VNTR 47) had the same number of tandem repeats in all 90 F57- and IS*900*-positive samples. Based on the results of this study, locus VNTR 3 seemed to differentiate S-type from C-type MAP revealing one tandem repeat for all three S-type MAP samples, in contrast to two tandem repeats for all remaining 87 C-type samples.

MIRU-VNTR typing is a simple and reproducible typing system, which can be used also for samples with a low amount of DNA allowing for high-throughput screening (30, 31). MIRU-VNTR typing can however overestimate or underestimate the relationship between strains (9, 16, 32), possibly due to the instability of certain genomic repeats or horizontal gene transfer (9). Homoplasy is the occurrence of genotypes that are identical by state but not by descent and can appear due to different reasons, such as convergent and reverse evolution or horizontal gene transfer (33), possibly interfering with the interpretation of data derived from VNTR analysis. In a recent study of Byrne et al., the presence of multiple distantly related strains within individual farms was shown, suggesting different infection events with distinct MAP strains (7). No clonal isolates were identified on the same farm revealing each isolate to have its own pattern of variants (7). In our study, intra-herd variability was observed in four out of 11 herds, of which multiple samples per farm were analyzed. Another recent study analyzing MAP by WGS showed that MIRU-VNTR typing lacks resolution for fine tracing of MAP strain circulation between and within herds (15). Despite these limitations, MIRU-VNTR typing, in combination with the differentiation between C-type and S-type MAP including subtype identification, can disclose an interesting insight about the strain variability in view of a temporal or geographical distribution of INMV types and the proportions of C- and S-type MAP strains. A study from New Zealand showed higher numbers of S-type strains compared to C-type strains among beef cattle (19), in Europe however, S-type MAP was not as frequently found in cattle. In the present study, S-type MAP was detected in three samples of bovine feces without cultivation. One S-type sample was collected from a Brown Swiss cow of a dairy farm, whereas the two other S-type samples originated from farms involved in beef production collected from cattle of the breeds Piemontese and Charolais, respectively. In Switzerland, cows frequently spend the summer on alpine pastures shared by different cattle herds and wildlife, giving animals plenty of occasions for co-grazing with other ruminants. During wintertime, the animals usually go back to their farm in the valley. This kind of animal movement favors the transmission of different MAP strains between herds and wildlife. In a previous study, morphological and molecular characterization of a new S-type III MAP strain (INMV 218) was described in goats (20). In comparison to the herein identified S-type III MAP types INMV 72 and INMV 253, differences could be observed in locus VNTR 7, finding an imperfect repeat of 1.5 for the earlier analyzed caprine samples versus two and one tandem repeats for profiles INMV 253 and INMV 72, respectively. Locus MIRU X3 had one repeat for INMV 72, while the caprine and bovine S-type profiles (INMV 218 and INMV 253) included two tandem repeats.

Since MAP, and especially S-type MAP, has a slow growth rate and is very tedious to cultivate, it is an advantage to have a molecular tool, which recognizes strain diversity from bovine feces without prior cultivation. Studies based on WGS can provide reliable information about phylogenetic relationships, however, due to the laborious cultivation of S-type MAP in the laboratory, many circulating strains might possibly stay undetected. Further studies will be needed to correlate different MAP genotypes with morphological lesions in the bovine intestine and/or the clinical relevance.

## Data availability statement

The original contributions presented in the study are included in the article/Supplementary material, further inquiries can be directed to the corresponding author.

## Author contributions

MR-H, MB, RS, FS, and SS designed the study and were involved in formal data analysis. MR-H, SS investigated the data. MR-H and SS wrote the original manuscript. MB, RS, and FS reviewed and edited the manuscript. All authors contributed to the article and approved the submitted version.

## Conflict of interest

The authors declare that the research was conducted in the absence of any commercial or financial relationships that could be construed as a potential conflict of interest.

## Publisher’s note

All claims expressed in this article are solely those of the authors and do not necessarily represent those of their affiliated organizations, or those of the publisher, the editors and the reviewers. Any product that may be evaluated in this article, or claim that may be made by its manufacturer, is not guaranteed or endorsed by the publisher.

## Footnotes

^1^ http://mac-inmv.tours.inra.fr/
